# Partnership, living arrangements, and low birth weight: evidence from a population-based study on Spanish mothers

**DOI:** 10.1186/s12884-022-05263-0

**Published:** 2022-12-09

**Authors:** Chiara Dello Iacono, Miguel Requena, Mikolaj Stanek

**Affiliations:** 1 Department of Sociology and Communication, University of Salamanca, Salamanca, Spain; 2 Department of Sociology II, UNED, Madrid, Spain

**Keywords:** Birth weight, Spain, Extended households, Family structure, Perinatal health

## Abstract

**Background:**

Birth weight is considered a crucial indicator of individual and population health, as it determines a newborn’s growth and development. An extensive body of research has explored various determinants of perinatal health, including the impact of living arrangements. This population-based study analyzes the relationship between mothers’ partnership status and household structure and children’s low birth weights. It addresses two basic research objectives: on one hand, how living/not living in a couple affects birth weight; on the other, how partnership status impact on birthweight when mothers live in extended households with other non-nuclear members.

**Methods:**

A novel database provided by the Spanish Office for National Statistics (INE), which links the 2011 census with births registered from 2011 to 2015 (sample size 22,433) is used. Llogistic regression models are estimated tto obtain adjusted odds ratios (OR) for the relative effects of living arrangements and other covariates such as characteristics of births and mothers’ socioeconomic profiles, on birth weight.

**Results:**

Differences in low-birth-weight rates may be attributed to the dissimilar socio-demographic characteristics of the groups of mothers in the different coresidential situations. Although our models revealed that the impact of the covariates on birth weight was similar to that shown by previous studies, this was not the case for the effect of the main explanatory variable. Contrary to expectations, the presence/absence of a male partner in nuclear or in extended households does not reveal significant protection against low birth weight. Children born in households in which the male partner was absent were not more likely to have a low birth weight. On the other hand, analyzing the possible protective effect of extended households, we did not detect significant differences in the likelihood of low birth weight between single mothers without and with non-nuclear coresidents in their households.

**Conclusions:**

Our analysis provides novel evidence regarding the effect of partnership status and household type on perinatal health in Spain. First, contrary to what has been observed in previous studies in Spain and elsewhere, our study shows that living without a partner has no effect on low birth weight. Second, we reveal that households including non-nuclear coresidents are associated with low birth weight suggesting that even in a basically familist societal context such as the Spanish one, the extended family does not fully protect against poor perinatal outcomes.

## Introduction

Two interesting lines of research have recently connected the birth weights of newborns and the structure of their mothers’ households. First, birth weight is considered a crucial indicator of individual and population health, as it determines the likelihood of a newborn going on to experience satisfactory growth and development [1, 2]. Low and very low birth weights, respectively < 2500 and < 1500 g, are associated with increased risk of neonatal mortality [3] poorer health status in adulthood [4] and specific morbidities such as hypertension, type 2 diabetes, coronary heart disease, metabolic syndrome, and obesity [5]. The negative impact of low birth weight on an individual’s development has also been highlighted in terms of its consequences for neurological maturity and its relationship to intellectual and cognitive functions [6, 7], educational attainment [8] and socioeconomic status in adulthood [9–12] low birth weight remains a major public health problem [13] and low-birth-weight rates have increased in southern European countries in recent years [14], it is necessary to understand the different factors that influence this aspect of perinatal health.

Second, the demographic events of recent decades and their structural, cultural, and social consequences have led to new family patterns differentiated in terms of cohabitation, household type, size, and composition [15–17]. In Spain, households have recently experienced numerous changes in their internal composition, including the decline of the so-called “traditional” family made up of a mother, a father, and biological children [17] and the development of new forms of domestic coresidence [18]. Over the last few decades, an increase in the share of non- traditional households, such as single-person households, male and female single-parent households, reconstituted families, multiple families, non-nuclear households, and other living arrangements has been observed [17, 19, 20]. In particular, the rise of single-parent households composed of single women with children has increased the social visibility of lone motherhood and out-of-wedlock births [18]. Moreover, recent studies have shown that in Spain, unlike in Italy, the covariation between educational attainment and single motherhood is negative [21] and this negative gradient is the result of a reversal since the early 1990s [22].

Previous studies in various contexts have connected the research on birth weight and household composition by suggesting that household type may be an important factor in children’s birth weight [23]. This research has addressed the effect of living arrangements on birth outcomes primarily in terms of the partnership status of the mother, focusing on the presence/absence of partners in the household. Partnered mothers tend to give birth to healthier and heavier children because the two-parent household provides support throughout the woman’s pregnancy, as domestic and economic functions can be shared by both parents [24] however, mothers living alone lack this source of support and are more exposed to perinatal health risks such as neonatal mortality, low birth weight, and the development of specific morbidities at the time of childbirth [25–27]. Studies have highlighted that the marital bond represents protective factor for the perinatal health of children: if mothers live alone, their children’s risk of low birth weight is higher [18, 23, 28]. Moreover, some single mothers turn to the extended family network for help with the vulnerability they experience. In such cases, a more complex household structure is established in which members outside the nucleus are supposed to provide the support the mothers need [26].

Against this background, it is expected that coresidence with a partner will have a positive effect on perinatal health indicators, specifically on birth weight: cohabiting mothers will benefit from the protective effect of partnership and partner support in avoiding poor perinatal health outcomes and will have fewer low-birth-weight babies than non-cohabiting mothers. In addition, some single mothers may join an extended household as a specific strategy to alleviate difficulties during and after pregnancy. It can be assumed that the family solidarity of the extended household will reduce the incidence of low-birth-weight children, particularly among non-partnered mothers. The protection of the extended household for single mothers is even more plausible given the proven negative educational gradient of single motherhood in Spain [21, 22]. If single motherhood is more likely to occur among the low socioeconomic status segments, it is to be expected that they would more actively seek family support in the extended household. In other words, coresidence with extended household members may moderate the effect of mothers’ lack of a partner on low birth weight.

These expectations led us to formulate four hypotheses. H1: Lacking protection and support from a partner, lone mothers who do not live-in nuclear households have higher rates of low-birth-weight children than women living with a partner do. H2: Because of the protection and support deficit resulting from the lack of a partner, even when supported by an extended household, non-partnered mothers living with non-nuclear coresidents have children with higher rates of low birth weight than partnered women living with non-nuclear coresidents. H3: Non-partnered mothers who live with non-nuclear coresidents have lower rates of low birth weight than non-partnered women who do not live-in extended households. H4: Partnered mothers living in extended households have lower rates of low birth weight than partnered women in nuclear households because the protective effects of a partner and extended household are additive.

## Methods

### Data and sample

Our data come from a novel database provided by the Spanish National Statistics Office (Instituto Nacional de Estadística, INE) that links the 2011 census, which contains information on individuals’ personal characteristics such as gender, age, marital status, country of origin, education level, employment status, living conditions, migration status, and type of household, with information about births registered between January 2011 and December 2015 and vital statistics from the Spanish MNP. The whole dataset represents a sample of approximately 10% of the Spanish population. Individual birth data from the years 2011 and 2012 for mothers aged 15 to 49 living in different types of households were used for this population-based study. As information on household structure is available for the year 2011, the observation period is limited to only two years (2011 and 2012) to avoid possible changes over time in household structure undermining the reliability of the measure. Three exclusion criteria were applied to construct the analytical sample. First, multiple births were excluded since it is known that they have a different intrauterine growth pattern from gestational weeks 28–30. Second, observations with missing information in the relevant variables such as birthweight and gestational age were discarded. Third observations with impossible combinations of birthweight and gestational age based on references published elsewhere [29] were not considered. The final analytical sample included 22,433 observations. According to the Spanish Organic Law 3/2018 on the Protection of Personal Data and the guarantee of digital rights this kind of population-based study (anonymized national data) did not require ethical approval.

### Variables

Our dependent variable is low birth weight, which is set below 2500 g according to the threshold commonly adopted in the literature [5]. The household structure recorded in the 2011 Spanish census is the main explanatory variable. To test our hypotheses, we conducted a four-way comparison. First, we determined four types of household according to whether the mother lives with a partner or non-nuclear coresidents: (*a*) mothers without a partner and without coresidents outside the nucleus; (*b*) mothers without a partner but with coresidents outside the nucleus; (*c*) mothers with a partner but without other coresidents outside the nucleus; and (*d*) mothers with a partner and with coresidents outside the nucleus. Our analysis includes available covariates that are commonly used as controls in regression models for birth weight. The sex of the newborn is included because on average, girls weigh less than boys [5, 30]; birth order (first, second, or third successive birth) is included because firstborn children tend to be smaller than successive children [5, 29] gestational age—preterm (23–36 weeks) or normal (37–39 weeks) as a mediator variable in the sense that certain household types may act as stressors that accelerate delivery and produce low birth weight. Mother’s age at delivery [31, 32] grouped into three categories (under 25 years, between 25 and 34 years, and 35 years or older) and whether the mother was born in Spain or is an immigrant [29] were also included. Our socioeconomic measure is educational attainment divided into three categories (primary education or below, secondary, and tertiary).

### Analytical strategy

Our analytical strategy is based in a four-way comparison. Comparison of the birth weights of children born in type *a* and *c* households indicate the effect of not having a partner on birth weight among mothers living without non-nuclear coresidents to test H1. Comparing types *b* and *d* shows the effect of not having a partner on birth weight among mothers living with non- nuclear coresidents, testing H2. Comparison of types *b* and *a* shows the effect on low birth weight of unpartnered women living with non-nuclear household members, testing H3. Finally, by comparing types *d* and *c*, we can estimate the effect of coresidence with non-nuclear partners among women with partners and test H4. The empirical analysis proceeds as follows. After determining the basic descriptive results of the studied population and assessing the total effects of living arrangements on low birth weight, we estimated logistic regression models to obtain adjusted odds ratios (OR) for the risk of giving birth to a low-birth-weight baby. The first model uses the entire analytical sample and includes household type as the focal independent variable, in addition to other covariates that need to be controlled to gain precision and avoid potential confounding factors. Four other logistic regression models are estimated that compare, two by two, in the manner described above, the birth weight outcomes of children born to mothers in different living arrangements. These four models allow separate estimations of the effects of a partner and other non-nuclear coresidents on birth weight. In addition to logistic regression models, we post-estimated average marginal effects to circumvent the possibility that the OR analysis reflects unobserved heterogeneity, i.e., the influence of omitted factors impacting on our dependent variable. As indicated by Mood (2010) [33] comparing ORs between groups can be problematic if the unobserved heterogeneity varies from one group to another. In the present case, omitted variables could differentially affect birth weight depending on the type of household. Average marginal effects provide an acceptable solution to the problem of unobserved heterogeneity when comparing household type outcomes within the same sample. All the models have been estimated using Stata 15.

## Results

Table 1 presents the results of the descriptive analysis of the sample according to the mothers’ living arrangements. In general, the distribution of mothers of different ages in different types of household corresponds to the distribution of different forms of coresidence in Spain. One- couple households are by far the predominant type over other forms of coresidence, such as single persons, single-parent families, and complex households that include non-nuclear coresidents. Data show that three out of four women in our sample (75%) were living with a partner and with or without children, and with no non-nuclear coresidents, whereas only 7.2% lived with a partner and coresidents from outside the nuclear family. Similar proportions were observed for mothers living without a partner. Interestingly, more mothers who were not living with a partner were living with non-nuclear coresidents (10%) than without them (7.8%).

**Table 1:** Descriptive Statistics

Mothers without partner					Mothers with partner			
	Without non-nuclear coresidents		With non-nuclear coresidents		Without non-nuclear coresidents		With non-nuclear coresidents	
	%	N	%	N	%	N	%	N
Year of birth								
2011	45.2	786	39.9	896	52.1	8761	53.5	870
2012	54.8	954	60.1	1351	47.9	8059	46.5	756
Low Birthweight	9.7	168	11.4	256	8.5	1433	10.2	166
Sex of new born								
Male	54.0	939	51.5	1158	51.9	8732	52.4	852
Female	46.0	801	48.5	1089	48.1	8088	47.6	774
Birth Order								
1	51.3	893	73.0	1640	40.1	6740	41.8	680
2	42.1	733	23.3	523	49.7	8354	45.7	743
3+	6.6	114	3.7	84	10.3	1726	12.5	203
Gestational Age								
Normal	91.0	1584	88.5	1988	91.1	15,329	90.3	1468
Premature	9.0	156	11.5	259	8.9	1491	9.7	158
Age of Mother								
< 25	1.8	31	29.6	666	2.2	364	13.1	213
25–34	55.2	960	49.4	1110	56.7	9537	54.1	880
35+	43.0	749	21.0	471	41.1	6919	32.8	533
Education of Mother								
Primary or Less	22.2	387	44.5	999	23.5	3946	39.4	640
Secondary	34.8	606	29.7	668	33.1	5574	32.9	535
Tertiary	42.9	747	25.8	580	43.4	7300	27.7	451
Origin of Mother								
Native	92.0	1601	86.5	1943	90.2	15,172	68.1	1107
Immigrant	8.0	139	13.5	304	9.8	1648	31.9	519
Total	7.8	1740	10.0	2247	75.0	16,820	7.2	1626

Compared to the other categories, mothers residing in typical nuclear households, with a partner and without non-nuclear coresidents, gave birth to proportionally fewer children with a low birth weight (8.5%), followed by mothers without a partner and without non-nuclear coresidents (9.7%). Mothers without a partner who were living with non-nuclear coresidents.

had the highest rate of low-birth-weight babies (11.4%), surpassing the rate for mothers with a partner and with non-nuclear coresidents (10.2%). As shown in Table 1, the differences in birth weights between mothers in different living arrangements were small. The descriptive analysis of the unadjusted risks of low birth weight points to (i) a positive but rather small impact of non-partnership (+ 2.0% = [(9.7*168 + 11.4*256)/424] [(8.5*1433 + 10.2*166)/(1599)]) and (ii) another small positive risk of low birth weight of living with non-nuclear coresidents (+ 2.3% = [(11.4*256 + 10.2*166)/422] − [(9.7*168 + 8.5*1433)/(1601)]). In this risk difference metric, the associations of living without a partner and low birth weight are the same for single mothers living in nuclear households (1.2% = 9.7% − 8.5%) and those living in extended households (1.2% = 11.4% − 10.2%). The risk of low birth weight among mothers living with non-nuclear coresidents does not depend on partnership status: it is the same for partnered mothers (1.7% = 11.4% − 9.7%) as for non-partnered mothers (1.7% = 10.2% − 8.5%). The observed differences in low-birth-weight rates may be due in part to the compositional heterogeneity of the groups of mothers in different living arrangements. As shown in Table 1, the socio-demographic characteristics of the groups of mothers in the different coresidential situations were very dissimilar. Age, for example, was an important differentiating characteristic. Non-partnered mothers living with non-nuclear coresidents not only had the highest proportions of underweight children but were also by far the youngest of the four groups, as nearly 30% of them were under 25 at the time of delivery. The proportion of young mothers under the age of 25 living without non-nuclear coresidents, both partnered and unpartnered, was around 2%. Finally, those who are partnered and living with co-residents constitute 13% of all women under the age of 25.

In addition, we can see that the youthfulness of young mothers living without a partner and with non-nuclear coresidents was associated with higher rates of firstborn children, premature gestational age, and lower educational attainment, all factors known to be associated with low birth weight. As Table 1 shows, 44.5% of the non-partnered mothers living with coresidents had only primary-level education or lower, a low educational profile that is only approached by partnered mothers living with non-nuclear coresidents (39.4%) and which distinguishes them from non-partnered and partnered mothers living without non-nuclear coresidents (22.2 and 23.5%, respectively, with primary education or less). Finally, the heterogeneity of the migrant status of one of these groups of mothers should also be highlighted: 32% of the partnered mothers with non-nuclear coresidents were immigrants, which clearly differentiated them from the other groups of mothers (with between 8 and 14% immigrants).

**Fig. 1:**
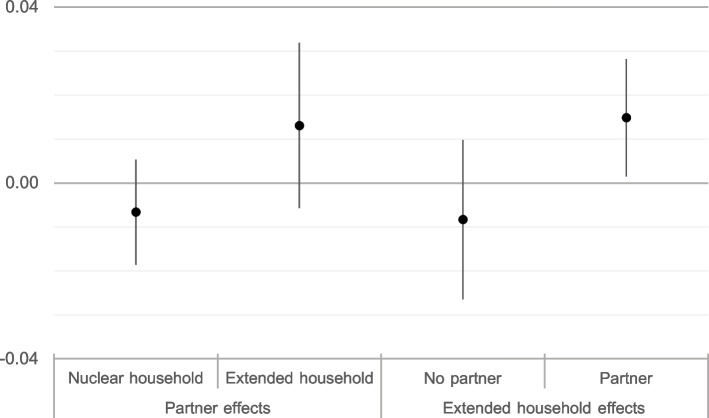
Average marginal effects of partnership status and household structure on low birth weight, with 95% Confidence Intervals. Estimated from models 1 to 4 in Table 3

Table 2 shows, in terms of OR, the unadjusted simple and combined effects of not living with a partner and living with extended household members. Not living in a couple and living in extended households imply that the likelihood of a low birth weight for children of mothers living in these situations is higher than that for children of partnered women and those living in nuclear households. However, although statistically significant, these effects are well below the so-called recommended minimum effect size representing a practically significant effect for social science data [34]. Moreover, when the impact of living/not living with a partner were estimated separately for nuclear and extended households, the associations retained their positive sign but were no longer significant. Living with non-nuclear coresidents had also small influence among both partnered and non-partnered mothers and was barely significant for the former. Table 3 shows the estimates of a series of logistic regression models to analyze the relative importance of living arrangements, as well as other characteristics of births and mothers’ socioeconomic profiles, for birth weight. The results of Model 0 (the whole sample) revealed that the associations of mothers’ living arrangements and low birth weight were small and non-significant when controlling for possible confounding factors. Compared to mothers living without a partner or non-nuclear coresidents, mothers in different living arrangements did not have a significantly different probability of having a child with a low birth weight; their coefficients were very close to the value of the reference category and their values fell within the range of what can be expected from sampling variability. In terms of birth characteristics, as expected, lower birth weights were observed for girls and firstborn children. In relation to the mother’s migration status, even though immigrant women had fewer socioeconomic resources, they had better levels of gestational health than women born in Spain. Finally, the expected educational gradient of low birth weight was found: the higher the mother’s educational level, the lower her probability of giving birth to a low-birth-weight baby. The remaining models in Table 3 show the estimates of ORs for low birth weight for the four comparisons between mothers in different living arrangements. The two-by-two comparisons in Models 1 to 4 showed that the known associations of birth characteristics and mothers’ characteristics were very consistent. The ORs estimated for birth characteristics (sex of newborn, birth order, gestational age) and mother’s characteristics (age, educational attainment, migration status) in the four comparisons between different living arrangements varied with the expected intensity and in the expected direction. However, the ORs measuring the impact of living arrangements on low birth weight were close to 1, pointing to no significance. For example, Model 2 compared non-partnered and partnered mothers in extended households and showed a higher probability of low birth weight among the children of the latter (OR = 1.206). This higher probability was contrary to our expectation, but the effect was not statistically significant. Model 3, which compared non-partnered mothers living in nuclear households with non-partnered mothers in extended households, estimated a lower probability of low birth weight for the children of the latter (OR = 0.882), as expected, but since the OR was not significantly different from 1, it cannot be said that the support of the extended household compensated for the negative impact of living without a partner. Only Model 4, which compared partnered mothers living in extended households with those living in nuclear households, showed a positive and significant impact (OR = 1.28) of coresidents on the likelihood of low birth weight. In other words, all other factors being equal, living with non-nuclear coresidents appeared to increase, not decrease, the likelihood of partnered mothers having low-birth-weight children. It is notable that the OR of Model 4 was not only significant but also greater than 1 and therefore contrary to H4. Partnered mothers living in extended households had a higher risk of low birth weight than partnered mothers in nuclear households. In other words, the protective effect of coresidents did not add to the protective effect of living with a partner. The average marginal effects on low birthweight for each household type, estimated form models 1 to 4 in Table 3, are shown in Fig. 1. Estimated AEs fully confirm the results of the ORs: they are substantively and statistically insignificant. The bars of the confidence intervals include the value 0 except for women with partners living in extended households who are slightly more likely to have low birthweight children than women with partners in nuclear households.

**Table 2:** Unadjusted effects of partnership status and living arrangements of mother on low birthweight

	Effect	OR		SE	95% Conf. Interval	
Type of households						
All households	No partner	1.254	***	0.072	1.120	1.404
Nuclear households	No partner	1.148		0.098	0.970	1.358
Extended households	No partner	1.131		0.119	0.920	1.390
Partnership status						
All mothers	Non-nuclear coresidents	1.295	***	0.075	1.157	1.451
Partnered mothers	Non-nuclear coresidents	1.221	*	0.106	1.031	1.446
Non-partnered mothers	Non-nuclear coresidents	1.203		0.126	0.980	1.478

**Table 3:** Effects of selected variable on low birthweight. Full sample (Model 0) and four comparisons between different living arrangements

Model 0			Model 1		Model 2		Model 3		Model 4	
	OR	SE	OR	SE	OR	SE	OR	SE	OR	SE
Living arrangements										
No partner, without non-nuclear coresidents	ref.		ref.				ref.			
No partner, non-nuclear coresidents	0.916	0.119			ref.		0.882	0.123		
Partner, without non-nuclear coresidents	0.893	0.090	0.893	0.091					ref.	
Partner, non-nuclear coresidents	1.140	0.158			1.206	0.164			1.275*	0.135
Sex of new born										
Male	ref.		ref.		ref.		ref.		ref.	
Female	1.432***	0.079	1.390***	0.086	1.608***	0.200	1.527***	0.191	1.411***	0.087
Birth Order										
1	ref.		ref.		ref.		ref.		ref.	
2	0.578***	0.035	0.579***	0.038	0.578***	0.086	0.561***	0.085	0.580***	0.038
3+	0.550***	0.060	0.538***	0.064	0.638	0.171	0.658	0.209	0.536***	0.062
Gestational Age										
Normal	ref.		ref.		ref.		ref.		ref.	
Premature	0.036***	0.002	0.036***	0.002	0.038***	0.005	0.034***	0.004	0.037***	0.002
Age of Mother										
< 25	ref.		ref.		ref.		ref.		ref.	
25–34	0.980	0.119	0.972***	0.197	0.978	0.159	0.959	0.174	1.023	0.176
35+	1.182	0.151	1.144***	0.235	1.359	0.265	0.939	0.199	1.284	0.226
Education of Mother										
Primary or Less	ref.		ref.		ref.		ref.		ref.	
Secondary	0.860*	0.060	0.885	0.071	0.782	0.114	0.904	0.140	0.848*	0.066
Tertiary	0.664***	0.047	0.684***	0.054	0.585***	0.100	0.708*	0.119	0.655***	0.052
Origin of Mother										
Native	ref.		ref.		ref.		ref.		ref.	
Immigrant	0.670***	0.063	0.657***	0.075	0.702*	0.115	0.563**	0.125	0.698***	0.072
Constant	1.809***	0.288	1.853**	0.416	1.535**	0.254	1.999**	0.453	1.530*	0.272
Pseudo R square	0.271		0.270		0.270		0.288		0.265	
N	22,433		18,560		3873		3987		18,446	

Note. Model 0 includes the full sample. Model 1 compares non-partnered mothers without coresidents with partnered mothers without coresidents. Model 2 compares non- partnered mothers with coresidents with partnered mothers with coresidents. Model 3 compares non-partnered mothers without coresidents with non-partnered mothers with coresidents. Model 4 compares partnered mothers without coresidents with partnered mothers with coresidents

## Discussion

Birth weight is determined by multiple factors. Its main determinants may be biological. i.e., related to the sex of the baby, the weight of the mother, parity at birth, the presence of chronic maternal disease, complications during pregnancyand obstetric difficulties, or behavioral, relating to the habits of the mother [35]. Maternal age has also been found to influence the risk of low birth weight, both when pregnancy occurs at a young age [31] and later in life [32, 36] Several studies have suggested that socioeconomic status also influences birth weight [5, 30, 37, 38] Studies have emphasized the characteristics of the mother and/or father in terms of education, income, and employment status [36, 39–42]. Differences in the birth weights of children born to mothers with different origins or migration status have also been shown. For example, immigrant women in Spain have better perinatal health outcomes than natives [43–45]. An additional factor that can affect birth weight and that has lately gained increasing attention is the mother’s partnership status. In recent decades, the growth of single-parent households in many economically developed societies has spurred research on the social, economic, and demographic characteristics of women who choose single parenthood and how these relate to the perinatal health of their children [5, 28, 37, 39, 46]. One of the main focuses has been the association of mothers’ partnership status and the birth weight of their children [27, 28, 47]. According to previous research, the children of partnered mothers (those who are married and/or cohabiting with a partner) have better birth weight outcomes than those of unpartnered mothers. Women who live with a partner generally have access to more resources, healthier prenatal practices, and the protection of a partner [24, 48]. It is assumed that the presence of a partner, regardless of formal marital status, improves the economic conditions of families, reduces the emotional stress experienced by the mother during pregnancy, and minimizes the risk of low birth weight and preterm birth [48]. In contrast, a lack of parental support or a stable relationship has been shown to lead to adverse pregnancy outcomes [47, 49]. Indeed, single mothers face a greater number of risk factors that can lead to low birth weight [28, 50], preterm birth [30, 51, 52], and even fetal or infant death [27, 53]. In countries such as Spain, where women with low levels of education are more likely to become lone mothers, single motherhood concentrates an accumulation of risks derived from their low socio-economic status and family situation [22].

This study analyzes the associations of partnership status and living arrangements with the birth weight of babies born in Spain between 2011 and 2012. . Data analysis does not reveal that lack of a partner has a substantive impact on low birth weight in nuclear or in extended households. Children born in households in which the male partner was absent were not more likely to have a low birth weight. The unadjusted ORs were small and not statistically significant and do not back our H1 and H2. Adjusted ORs estimated by logistic regression models (Table 3, Models 1 and 2) and post-estimated average marginal effects (Fig. 1) confirm this pattern. After controlling for some characteristics of births and mothers, we did not find a protective effect of male partners against low birth weight. Thus, our results provided novel empirical evidence contrary to longstanding research findings that single mothers were at greater risk of giving birth to low-birth-weight children [18, 24, 28, 48]. The Potential protection of extended households against low birth weight has also been analyzed. We hypothesized (H3) that among women without a partner, the family solidarity of the extended household reduced the incidence of low- birth-weight children. This conjecture was based on extensive empirical material indicating that some non-partnered mothers resorted to the extended household to alleviate difficulties during and after pregnancy [19, 54–57]. We also assumed that the protection of an extended household would be of particular significance in the Spanish cultural context, which is usually considered highly family-oriented [58, 59] and where single motherhood is more prevalent among low socioeconomic status strata [21, 22] Nevertheless, we did not detect significant differences in the likelihood of low birth weight between single mothers without and with non-nuclear coresidents in their households. Similarly, our expectations regarding the positive effect of family solidarity in extended households for partnered mothers (H4) were not confirmed. Contrary to expectations, our results indicated that all else being equal, partnered women living in extended households were more likely to have children with a low birth weight than partnered women living in nuclear households. Interestingly, this unforeseen association was statistically significant. In other words, greater complexity/extension of the households in which couples lived seemed to have a negative impact on the health of newborns as measured by birth weight. The effects of partnership and extended households were not additive. Although we do not have equivalent previous data to assess change over time, this null protective role of the extended household could suggest a considerable weakening of the non-nuclear family as a traditional provider of support to young mothers. Such a change would fit well with the socio-cultural transformation of the family in Spain that has taken place in recent decades [59, 60]. However, another possibility, coherent with the low educational and socio- economic profile of the partnered mothers living in extended households and their youth, would point to a small segment of Spanish society selected for their socio-demographic (and possibly behavioural) characteristics to have low birth weight children. Since negative selection induced by these unfavourable characteristics for perinatal health will be outweighing the support of the partner or the extended household, public authorities should pay more attention to difficulties, issues, and rejection faced by young mothers living in extended households.

### Strengths and limitations

The interpretation of the results of our study should be considered in light of certain strengths and limitations. The first strength of the study is related to the richness of the data, which allowed us to analyze the differences in the rates of low birth weight among a population of women aged 15 to 49 years in different types of households who gave birth in Spain in 2011 and 2012. Our research took advantage of a novel source of information provided by the Spanish Office for National Statistics with linked data from the 2011 Population Census and Spanish Vital Statistics corresponding to 2011–2012. Our data source provided new evidence of the impacts of living with a partner and/or with non-nuclear coresidents on the probability of having babies with a low birth weight. These rich data allowed us to observe the protective effects of (male) partners against low birth weight in the context of the structure of the households in which they and their female mates lived, a factor rarely considered in this type of analysis. In terms of limitations, our data lacked a set of control variables that are important predictors of birth weight, such as maternal chronic conditions, nutritional status, infections, tobacco, alcohol or drug use, and exposure to poor environmental conditions. This limitation arises from the fact that the birth registry does not include potentially relevant data on maternal health behaviors, health system utilization, or health conditions. Data and methodological limitations also prevent exploration of the causal mechanisms influencing the compositional and behavioral disparities between certain categories of mothers. These issues constitute a promising new field for future studies of the impact of living arrangements on perinatal outcomes.

## Conclusions

Our analyses contribute to discussions on the determinants of low birth weight in many ways. First, our study challenges previous research findings in which the (male) partner is an important provider of resources and care with proven influence on the perinatal health of the child. Our results indicated that no such relationship exists in the Spanish case, a societal context where the growing prevalence of single-parent households and their social acceptance is no longer an issue. Second, research on the effect of family status on perinatal health has so far focused mainly on the impact of the mother living with or without a partner within a nuclear household on low birth weight; less attention has been paid to the impact of living arrangements beyond the predominant framework of the nuclear family on birth weight. Although numerous studies have argued that in Spain—and more broadly in southern Europe—the family is an important agent of care and protection, relatively little is known about the protective role of extended families on perinatal health. This study has broadened the framework of analysis by considering diverse household compositions. Our results suggest that the starting situation of lone mothers is so precarious that living with the extended family is not sufficient to mitigate poor perinatal health outcomes. This pattern is especially striking in the case of very young single mothers. Although some single mothers may rely on other family members for support, the extended family is not able to counteract the disadvantageous situation that pushed them to seek this support. In these cases, the support of an extended household does not compensate for the deficits caused by the lack of a partner. Of course, that these households may also become stressors due to their scarce resources (crowding, poverty) cannot be ruled out. Third, a similar pattern can be observed among partnered women, as the protection provided by extended households did not add to that of being in a couple. 

This finding might also be due to the fact that the initial disadvantage that led the partnered mothers to seek the protection of non-nuclear coresidents is not reduced by the support that they provide.

## Data Availability

Data supporting the results of this study are available to the public under request and provided by the Spanish Statistical Office (INE) and computing code is available upon request to the corresponding author.
